# Osteoarthritis Therapeutics: From Microenvironment Understanding to Regenerative Intervention—Current Advances and Future Perspectives

**DOI:** 10.1111/os.70422

**Published:** 2026-09-01

**Authors:** Yongbiao Meng, Shenghua Wu, Quanchun Liu

**Affiliations:** ^1^ Department of Orthopedics First People's Hospital of Linping District Hangzhou China

**Keywords:** exosomes, microenvironment, OA, renewable biomaterials

## Abstract

Osteoarthritis (OA) is a prevalent, chronic degenerative joint disease characterized by progressive cartilage degeneration and impaired joint function. Its rising global incidence poses a significant health and socioeconomic burden. Traditional therapies primarily offer symptomatic relief but fail to halt disease progression and are associated with notable limitations. The contemporary understanding of OA pathogenesis has evolved from a model of mechanical wear to one emphasizing systemic imbalance within the joint microenvironment, involving immune dysregulation, metabolic disturbances, cellular senescence, and the gut‐joint axis. This paradigm shift supports the development of innovative, disease‐modifying strategies. This review is structured around a unifying multi‐axis microenvironmental imbalance model for osteoarthritis. It systematically integrates the crosstalk and cascade‐amplifying mechanisms of immune dysregulation, cellular senescence, metabolic disturbances, and the gut–joint axis, clarifying that multi‐dimensional microenvironmental disruption collectively forms the pathological core of OA. We focus on how renewable biomaterials, engineered exosomes, and their composite systems target and regulate the pathological network to restore joint homeostasis, enabling a paradigm shift from symptomatic management to root‐cause intervention. This work provides an integrated theoretical and translational roadmap for next‐generation disease‐modifying OA therapies.

## Graphical abstract

This article outlines a paradigm shift in OA therapeutics, OA as a systemic disease of joint microenvironment imbalance. It synthesizes advances in targeting key pathological hubs—immune, metabolic, and senescent—and details novel regenerative strategies using engineered biomaterials and exosomes. The review emphasizes the emerging potential of smart, integrated material‐exosome systems for spatiotemporally controlled, multi‐targeted therapy, paving the way for next‐generation, disease‐modifying interventions.

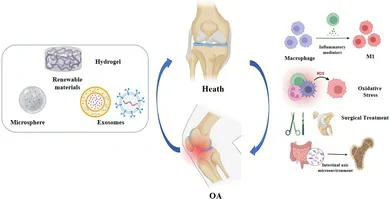

## 1 Introduction

Osteoarthritis (OA) is a chronic degenerative joint disease primarily characterized by the progressive breakdown of articular cartilage, leading to pain, stiffness, and functional impairment [1]. Symptomatic OA affects over 10% of the population aged 60 and above, with a higher prevalence in women, particularly in the knee and hand joints. The global burden is substantial, with projections estimating 642 million cases of knee OA worldwide by 2050, imposing a significant economic and healthcare burden [2]. Effective management of OA remains a major clinical challenge, as no definitive cure exists. Current therapeutic strategies primarily aim to alleviate pain, improve joint function, and enhance quality of life. For example, topical non‐steroidal anti‐inflammatory drugs (NSAIDs) are recommended for mild‐to‐moderate localized pain [3]. Oral acetaminophen has minimal gastrointestinal side effects and no anticoagulant effects [4]. However, these pharmacological interventions are associated with limitations, including risks of adverse effects, potential tolerance, and uncertain long‐term efficacy for chronic management. Consequently, there is a pressing need to identify more suitable and effective therapeutic approaches for OA.

The pathogenesis of OA has evolved from the traditional mechanical wear‐and‐tear theory to a multi‐axis microenvironmental imbalance model. At its core, OA is a dynamic homeostatic collapse involving multiple joint tissues (cartilage, subchondral bone, synovium, ligaments, joint capsule) under the crosstalk of mechanical stress, inflammation, metabolism, senescence, and gut‐joint axis signaling [5]. The disruption of multiple microenvironments is the fundamental cause of osteoarthritis's treatment resistance. Mechanical injury triggers chondrocytes to release damage‐associated molecular patterns (DAMPs), activating the TLR/NLRP3 innate immune pathway and driving the upregulation of pro‐inflammatory factors including IL‐1β, TNF‐α, and IL‐6, which inhibit cartilage anabolic metabolism [6]. Simultaneously, inflammation promotes synovial hyperplasia and loss of synovial fluid lubrication function, stimulating abnormal neurovascular invasion into cartilage and triggering pain sensitization [7]. In this process, the NLRP3 inflammasome, complement system, and chemokines jointly mediate matrix degradation, synovitis, abnormal bone remodeling, and neuropathic pain. Importantly, immune dysregulation, cellular senescence, metabolic disturbances, and gut–joint axis signaling do not act independently but mutually activate and cascade‐amplify each other, forming the central network driving OA progression [8, 9]. Therefore, understanding the complex microenvironmental mechanisms of OA is fundamental to developing effective therapeutic strategies.

With advancements in biology, chemistry, engineering, and related disciplines, biomaterials have found extensive application in orthopedic treatment. These materials enable multifunctional integration—such as mechanical support, immune modulation, drug‐controlled release, and synergistic tissue regeneration—while achieving minimally invasive precision delivery and biomimetic design [10]. Simultaneously exhibiting high biocompatibility, multi‐environment responsiveness, and long‐term relief for OA. For instance, smart ROS/pH/enzyme‐triggered hydrogels achieve targeted drug delivery in inflammatory microenvironments, while gradient scaffolds concurrently repair osteochondral defects [10]. Recent research on exosomes has broken through the limitations of traditional OA treatments. As key mediators of intercellular communication, exosomes have garnered attention due to their low immunogenicity, high stability, and ease of engineering modifications. Studies reveal that natural exosomescarry miRNAs and proteins. By suppressing inflammation, promoting cartilage synthesis, regulating macrophage polarization to repair the joint microenvironment. Additionally, engineered gene editing enables loading of therapeutic miRNAs or surface‐modified targeting peptides [11]. Composite systems combining biomaterials and exosomes overcome the limitations of monotherapy, achieving microenvironment responsiveness, targeted delivery, and sequential regulation, supporting the paradigm shift from symptomatic relief to root‐cause repair in OA treatment.

Centered on the multi‐axis microenvironmental imbalance model, this review systematically summarizes the crosstalk among four key dimensions of the OA microenvironment: immunity, senescence, metabolism, and the gut–joint axis. We highlight cutting‐edge advances in renewable biomaterials, engineered exosomes, and their composite systems, and critically analyze the regulatory mechanisms, advantages, and translational bottlenecks of each strategy. This work aims to provide a theoretical foundation and translational direction for next‐generation precise, intelligent, and disease‐modifying OA therapies.

## 2 Search Strategy and Selection Criteria

Systematic electronic searches were performed across PubMed and Web of Science for publications published up to February 2026. Search keywords were combined using Boolean operators (AND/OR), encompassing osteoarthritis, joint microenvironment, immune dysregulation, cellular senescence, gut–joint axis, regenerative biomaterials, exosomes, biomaterial–exosome composites, and disease‐modifying therapy. Manual backward screening of reference lists from retrieved authoritative reviews was additionally conducted to capture potentially eligible missing literature.

### 2.1 Inclusion Criteria

1. Studies centering on the multi‐axis OA microenvironment or regenerative OA therapeutics, with experimental validation based on chondrocyte assays, standard DMM/ACLT OA animal models, or human clinical trials. Research investigating biomaterial–exosome co‐delivery systems must report three core datasets: scaffold fabrication, exosome loading, and in vivo articular repair outcomes. 2. Peer‐reviewed original research articles or high‐quality reviews, with ≥ 70% of records published between 2021 and 2026; landmark foundational studies released prior to 2020 were limited to ≤ 30% of total citations. Human clinical trials and in vivo animal investigations were prioritized, whereas purely in vitro mechanistic data were only retained as supplementary evidence. 3. Articles sourced from officially indexed academic journals, with low‐impact publications restricted to ≤ 5% of all references.

### 2.2 Exclusion Criteria

Records were excluded if they covered irrelevant themes (orthopedic trauma implants, material characterization without OA‐related biological validation), preprints, conference abstracts without complete raw data, manuscripts from predatory journals, redundant studies with no novel mechanistic insights, publications with mismatched citation logic, and outdated articles older than 10 years lacking landmark theoretical significance.

As shown in Figure 1, we identified 1081 records from PubMed (NCBI) and 1503 records from Web of Science Core Collection through structured literature searches, with a unified publication date range from February 1, 2016 to February 1, 2026. All retrieved records were exported, combined, and deduplicated. We then screened titles and abstracts to exclude records unrelated to the topic, followed by full‐text assessment against the inclusion criteria based on the review's themes (OA; Microenvironment; Renewable biomaterials; Exosomes). Conference abstracts, case reports, and articles inconsistent with the review scope were excluded. The final set of articles included in the review (*n* = 75).

**FIGURE 1:**
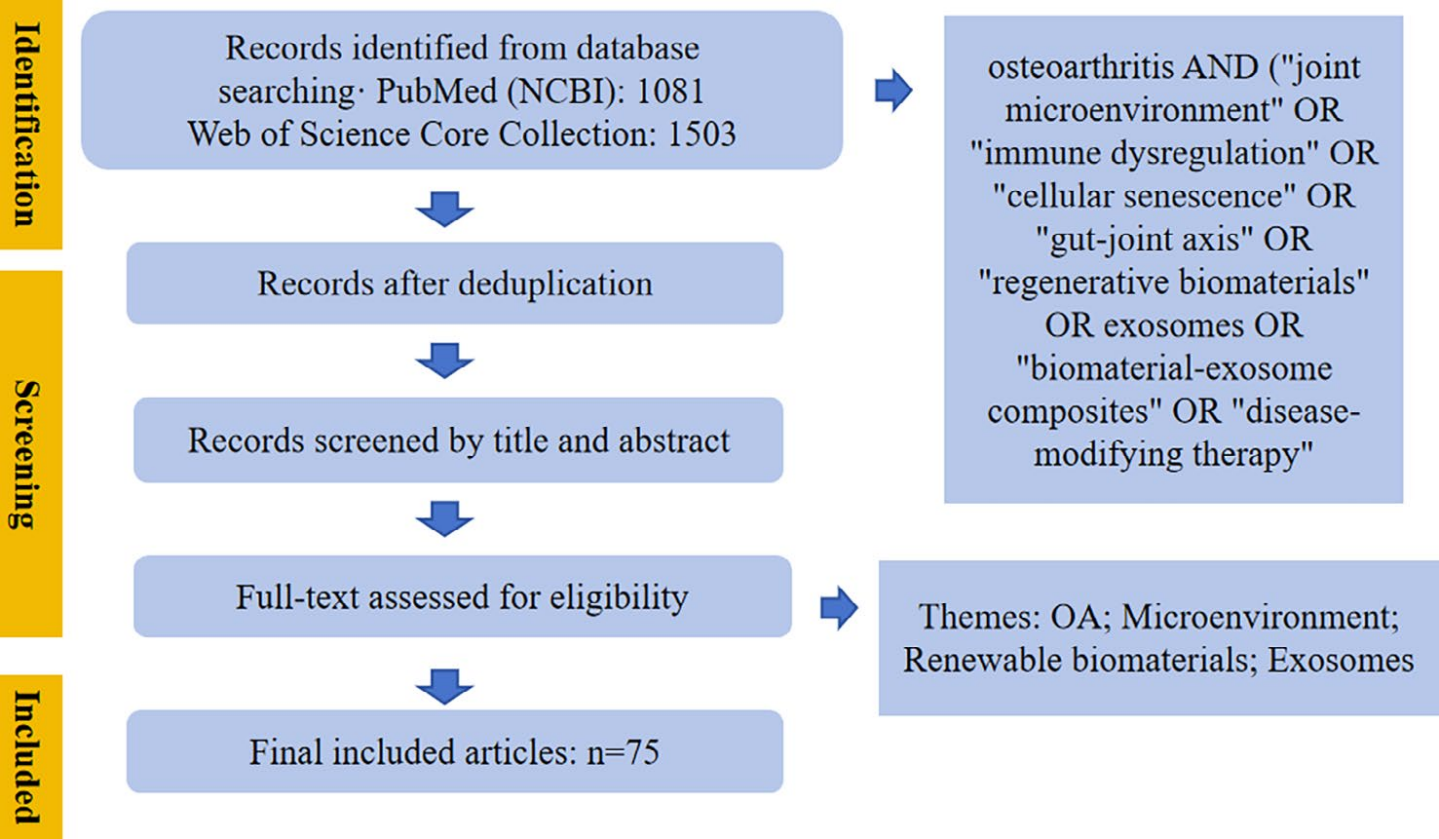
Literature selection flow diagram.

## 3 OA Overview

OA, also known as degenerative joint disease or osteoarthritis, is a chronic joint disorder characterized primarily by progressive degeneration of articular cartilage and secondary bone proliferation. Its typical features include joint pain, morning stiffness, limited joint mobility, crepitus during movement, and joint deformity in advanced stages [1]. It predominantly affects weight‐bearing or highly active joints such as the knee, hip, spine, and hands, representing a leading cause of disability in the middle‐aged and elderly, with incidence increasing markedly with age [12]. Traditionally, OA was considered to arise from the combined effects of mechanical and biological factors: aging, obesity, trauma, or overuse disrupt cartilage metabolic homeostasis, leading to loss of Type II collagen and proteoglycans, cartilage thinning and fragmentation, and impaired cushioning (Figure 2). These changes are accompanied by subchondral bone sclerosis, cyst formation, and marginal osteophytes, while synovial inflammatory mediators further exacerbate cartilage destruction [13].

**FIGURE 2:**
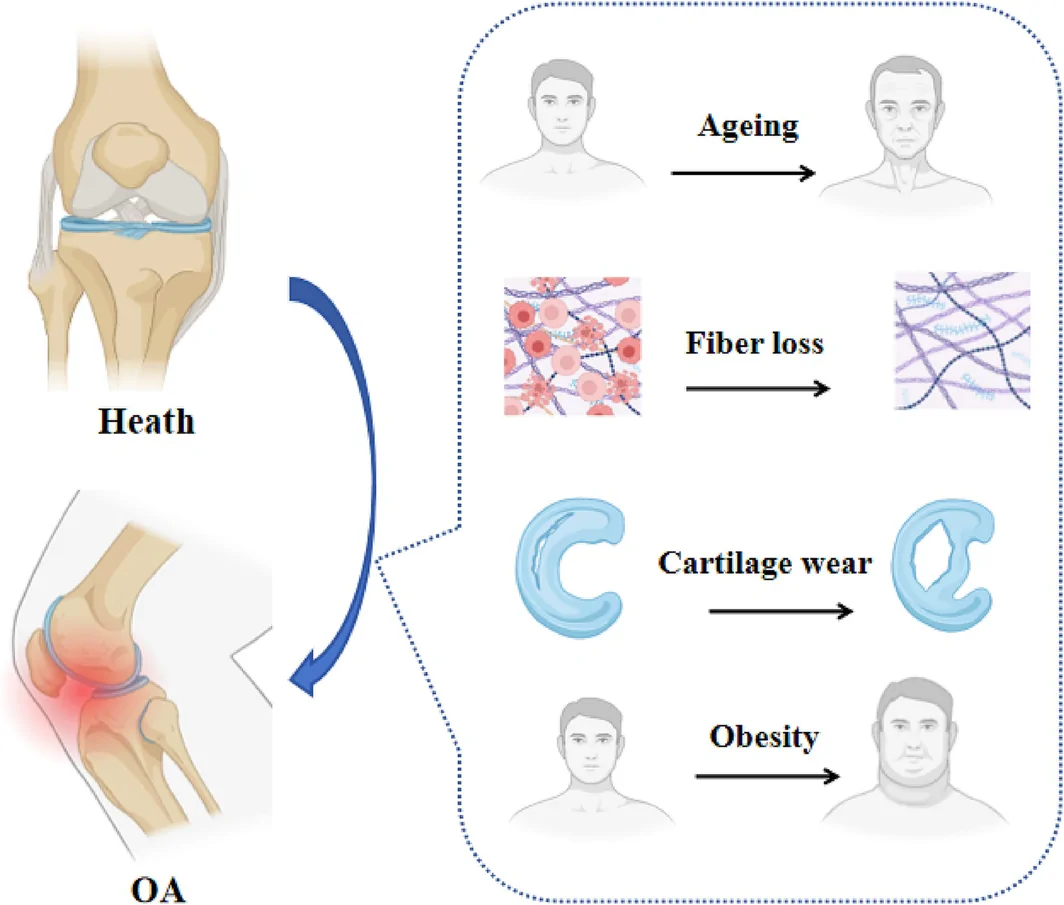
Causes of osteoarthritis.

In recent years, research has completely overturned the traditional view of OA as a purely local mechanical disorder, confirming that it is fundamentally a systemic disease driven by combined imbalance of the local joint microenvironment and systemic microenvironments. Within the bone microenvironment, an imbalance between synthesis and degradation of Type II collagen and proteoglycans in the extracellular matrix of articular cartilage marks the onset of cartilage degeneration. Increased activity of degradative enzymes such as matrix metalloproteinases accelerates the destruction of cartilage structure [14]. Synovial secretion of inflammatory factors including IL‐1β and TNF‐α establishes chronic local low‐grade inflammation [15]. Compensatory sclerosis and abnormal remodeling of subchondral bone affect superficial cartilage via vascular signaling [16]. Meanwhile, gut microbiota dysregulation regulates cartilage metabolic balance through the gut–joint axis by altering bile acid metabolism and GLP‐1 secretion [9]. Collectively, these findings demonstrate that OA is not a single‐tissue lesion but a complex disorder arising from coordinated disruption of the local microenvironment and systemic immune, metabolic, and neuroendocrine networks.

In summary, understanding of OA has evolved from local mechanical wear to a systemic theory of multi‐axis microenvironmental imbalance. Deepening insights into the microenvironment have not only redefined OA pathogenesis but also provided novel targets and strategies for innovative therapies targeting immunity, senescence, metabolism, and the gut–joint axis, driving the shift of OA treatment from symptomatic management to precise, root‐cause intervention.

## 4 Current Status of Mechanism‐Based Microenvironment Therapy

The pathological progression of osteoarthritis is driven by a coordinated imbalance of multiple microenvironmental axes, involving the crosstalk among immune dysregulation, cellular senescence, metabolic disorders, and gut–joint axis signaling. Targeting the joint microenvironment to correct pathological signals including inflammation, oxidative stress, abnormal bone remodeling, and neurovascular invasion can restore joint homeostasis, providing a theoretical basis for pharmaceutical, cellular, and biomaterial‐based therapies. This chapter systematically summarizes the current status of microenvironment mechanism‐based OA therapy and reveals the intrinsic connections and targeting strategies of each microenvironmental axis (Table 1).

**TABLE 1:** Current different treatment status of OA.

Classification	Detailed categories	Core strengths and critical limitations	Translational potential	References
Non‐pharmacological treatment	Extracorporeal shockwave, radiofrequency, acupuncture, laser therapy.	Non‐invasive; excellent safety; reduces local inflammation and pain; improves joint function; Cannot reverse cartilage degeneration.	Clinical routine; adjuvant therapy only.	[17]
	Operative treatment	Effective for end‐stage OA; significantly improves mobility and quality of life; joint replacement provides definitive structural correction; Highly invasive; surgical risk; prosthesis lifespan limited; cannot restore native joint microenvironment.	Clinical standard; end‐stage treatment.	[18]
Traditional drug therapy	Nonsteroidal anti‐inflammatory drugs	Rapid pain relief; improves knee function; low‐dose combination reduces systemic side effects; Short‐term symptomatic relief only; does not halt progression; long‐term use risks organ damage	First‐line clinical use; symptomatic only.	[3]
	Platelet‐rich plasma injection	Autologous origin; low immunogenicity; sustained clinical improvement for 6–12 months; promotes cartilage matrix synthesis; Easily affected by NSAIDs; high variability between preparations; no standardized protocol.	Widely used; needs standardization.	[19]
Microenvironment Response	Immune microenvironment	Targets M1 macrophage polarization; blocks TLR/NF‐κB signaling; restores cartilage anabolic/catabolic balance; Single‐target intervention; cannot regulate senescence/metabolic axes; short intra‐articular retention.	Preclinical; promising; needs optimization.	[20]
	Aging microenvironment	Eliminates senescent cells; inhibits SASP secretion; activates SIRT1‐mediated anti‐aging pathway; reduces cartilage destruction; Poor systemic delivery; off‐target effects; limited clinical safety data.	Early clinical; senolytics under trial.	[21]
	Metabolic fat microenvironment	Regulates lipid metabolism; inhibits AdipoR1‐NF‐κB inflammatory signaling; reduces obesity‐related OA progression; Slow onset; only effective for metabolic OA subtypes; no direct cartilage repair ability.	Preclinical; metabolic OA specific.	[22]
	Intestinal axis microenvironment	·Restores gut microbiota balance; inhibits systemic low‐grade inflammation; targets FXR‐GLP‐1 signaling for chondroprotection; Indirect regulation; slow onset; individual variability based on baseline microbiota.	Preclinical; systemic strategy potential.	[23]
Biomaterial Therapy	Collagen hydrogel	ROS‐responsive release; excellent injectability; promotes chondrogenesis; scavenges oxidative stress in early OA; Fast degradation; weak mechanical support; no multi‐axis microenvironment regulation.	Preclinical; early OA application.	[24]
	PLGA nanoparticle system	Cartilage‐specific targeting; severity‐dependent delivery; reduces cartilage degeneration and nociceptive pain; Complex preparation; limited payload capacity; short‐term efficacy.	Preclinical; targeted delivery potential.	[25]
	hUSCs‐140‐Exos	Highly stable miR‐140 delivery; activates mitophagy; restores subchondral bone structure; accelerates cartilage repair; High production cost; fast joint clearance; difficult to standardize; poor targeting.	Phase I/II; high potential; needs scaling.	[26]
	Spirulina platensis extracellular vesicles (SP‐EVs)	Plant‐derived; ultra‐low immunogenicity; inhibits JAK/STAT3; improves mitochondrial function; favorable biosafety; Low cargo loading efficiency; unclear long‐term efficacy; preclinical stage only.	Preclinical; safe but low potency.	[27]

### 4.1 Current Treatment

OA is a chronic joint disease characterized by degenerative changes in articular cartilage and bone proliferation. Its core pathological basis lies in irreversible damage to articular cartilage, manifested by reduced synthesis and increased degradation of collagen and proteoglycans in the extracellular matrix of chondrocytes. This leads to cartilage thinning and diminished elasticity, ultimately causing pain and functional impairment [1]. Aging, obesity, trauma, and chronic strain are major risk factors; obesity can increase knee joint pressure by 3–5 times [2]. Currently, no curative treatment exists for OA, and clinical care follows a stepwise comprehensive strategy: non‐pharmacological interventions (weight control, low‐impact exercise, physical therapy) serve as the foundation; pharmacotherapy is graded by pain severity: acetaminophen for mild pain, topical NSAIDs for moderate pain, and short‐term oral NSAIDs or opioids for severe pain [17]. Intra‐articular injection of hyaluronic acid or corticosteroids and oral glucosamine are also widely used, but their efficacy remains controversial [28, 29]. Collectively, all current treatments are symptomatic and cannot reverse microenvironmental imbalance or cartilage degeneration.

### 4.2 Microenvironment Response Therapy

#### 4.2.1 Immune Microenvironment

The microenvironment of the OA is a complex ecosystem shaped by multiple factors including immune cells, senescent cells, stem cells, adipose tissue, and distant microorganisms (Figure 3) [30]. The immune microenvironment in OA is centered on aberrant activation of macrophages, neutrophils, and chronic low‐grade inflammation [31]. In OA, macrophages can differentiate into pro‐inflammatory M1 and anti‐inflammatory M2 types. M1 macrophages secrete inflammatory cytokines (such as TNF‐α and IL‐1β), promoting cartilage degradation and inflammatory responses; M2 macrophages possess tissue repair and anti‐inflammatory functions, but their numbers and functions may be impaired in OA [32]. Based on this, researchers discovered that SHP2 is highly expressed in macrophages within the synovium of OA patients. Treatment with the SHP2 allosteric inhibitor SHP099 suppresses M1 macrophage polarization by inhibiting LPS‐induced TLR signaling. Concurrently, intra‐articular injection of SHP099 significantly attenuates OA progression [20]. Similarly, in OA, neutrophils release inflammatory mediators such as IL‐1β and IL‐6 along with elastase, activating matrix metalloproteinases (MMPs) to accelerate cartilage degradation. High neutrophil elastase (NE) expression inhibits chondrocyte proliferation, induces apoptosis, and blocks cell migration, accompanied by elevated intracellular reactive oxygen species and calcium levels. NE enhances caspase‐3 activity and disrupts mitochondrial membrane potential balance. Inhibitors significantly mitigate pathological processes in OA model rats [33]. Immune dysregulation is not an isolated event but an upstream core signal that triggers senescence and metabolic abnormalities, jointly forming a pathological amplification loop.

**FIGURE 3:**
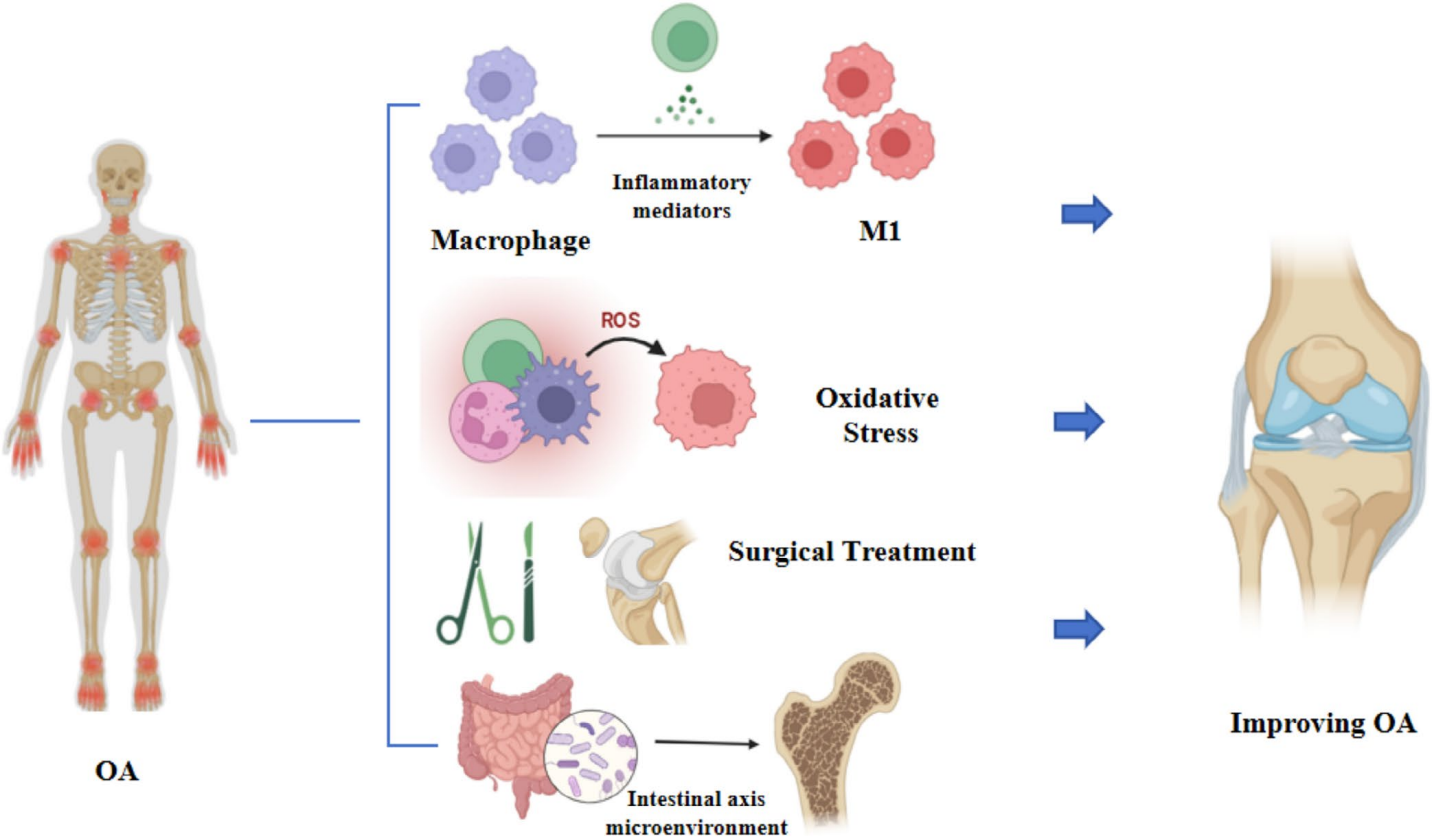
Therapeutic approaches targeting the microenvironment in OA.

#### 4.2.2 Aging Microenvironment

The core of the aging microenvironment is the senescence of chondrocytes, synoviocytes, and osteoblasts, which secrete the senescence‐associated secretory phenotype (SASP) including inflammatory factors, proteases, and ROS, impairing tissue self‐repair [34]. Aging chondrocytes reduce the synthesis of extracellular matrix components while increasing the secretion of matrix‐degrading enzymes, leading to cartilage degeneration. Research indicates that SASP, the most critical aspect of senescent cells, involves the secretion of a large collection of bioactive factors known as the senescence‐associated secretory phenotype. SASP includes pro‐inflammatory factors, chemokines, matrix metalloproteinases, growth factors, and others [35]. The number of senescent cells and expression of SASP factors significantly increased in OA joints and correlated with disease severity. Clearing senescent cells using senolytics—such as dasatinib plus quercetin combinations, nifuroxazide, or BCL‐2 inhibitors—effectively reduced joint inflammation, decreased cartilage destruction, and improved pain and function [36]. Early‐stage clinical trials for OA patients are currently underway, with preliminary results demonstrating promising safety profiles and potential therapeutic signals. Similarly, studies on AK/STAT inhibitors, NF‐κB inhibitors, metformin, and rapamycin/mTOR inhibitors have also shown the ability to suppress SASP production or signaling pathways, thereby exerting protective effects [37]. Notably, in the aging microenvironment, mitochondrial dysfunction, mitochondrial DNA damage, and impaired mitochondrial autophagy occur. This leads to excessive reactive oxygen species (ROS) production that overwhelms antioxidant defenses, resulting in oxidative stress [38]. ROS directly damages proteins, lipids, DNA, and extracellular matrix components, thereby activating pro‐inflammatory signaling pathways, inducing cellular senescence and apoptosis, and promoting MMP expression. Mitigation of OA progression through antioxidants or mitochondria‐targeted antioxidants, promotion of mitochondrial biogenesis, or enhancement of mitochondrial autophagy has yielded positive results in current preclinical models [21]. The aging microenvironment forms a positive feedback loop with the immune microenvironment, which is critical for OA chronicity and progression.

#### 4.2.3 Metabolic and Adipose Microenvironment

Intra‐articular fat pads (e.g., infrapatellar fat pad) are not only mechanical cushions but also immune and metabolic regulatory organs [39]. In OA, these adipose tissues undergo pathological activation, characterized by a significant increase in mesenchymal stem/progenitor cells, accompanied by enhanced angiogenesis and immune cell infiltration, collectively driving joint degeneration. Within the infrapatellar fat pad, a characteristic cellular subset—the mesenchymal inflammatory fibroblast—exists. This subset distinguishes the infrapatellar fat pad from subcutaneous and visceral fat, exhibiting pro‐inflammatory and pro‐proliferative properties. It influences chondrocytes via the MDK signaling pathway, contributing to osteoarthritis progression [40]. Oxidized low‐density lipoprotein can be taken up by macrophages in the synovium, activating inflammatory pathways and promoting ectopic bone formation. Similarly, hyperglycemia and insulin resistance promote the formation of advanced glycation end products [41]. These end products accumulate within articular cartilage, reducing collagen elasticity, increasing cartilage fragility, and upregulating inflammatory factor expression, thereby accelerating cartilage degradation. Research indicates statins may delay osteoarthritis progression in animal models and some observational studies. PPARγ agonists demonstrate potential for cartilage protection in preclinical studies by promoting cholesterol efflux and inhibiting inflammation. Similarly, metformin may exert chondroprotective effects by activating the AMPK/SIRT1 signaling pathway [42]. Additionally, studies in OA patients have shown that vitamin B1 supplementation alleviates osteoarthritis symptoms by inhibiting CCL2 secretion from macrophages. Increased intake of omega‐3 polyunsaturated fatty acids may exert a protective effect against OA through anti‐inflammatory mechanisms [43]. Metabolic disorders link the local microenvironment to the systemic system and represent the core driving mechanism of obesity‐related OA.

### 4.3 Gut‐Joint Axis

The gut‐joint axis is a key pathway for bidirectional local–systemic regulation in OA [44]. Specifically, patients with osteoarthritis often exhibit dysbiosis of the gut microbiota. This imbalance compromises the intestinal barrier function, allowing bacterial endotoxins to enter the bloodstream. This triggers systemic low‐grade inflammation, which in turn activates immune cells within the joints, accelerating cartilage destruction [45]. Meanwhile, abnormal bile acid metabolism reduces protective GUDCA, activates intestinal FXR, and decreases GLP‐1 secretion, weakening its anti‐inflammatory and chondroprotective effects [46]. A deeper understanding of the characteristics of the gut‐joint axis microenvironment has opened up a novel approach to treating osteoarthritis, shifting from traditional symptomatic pain relief to causal intervention, with targeting the gut emerging as a potential new strategy. Research reveals that low‐dose histamine derived from gut microbiota activates H3 receptors in the enteric nervous system, driving spinal microglia to shift from a pro‐inflammatory to a homeostatic phenotype. This establishes a novel pathway through the gut‐brain‐joint axis to regulate peripheral inflammation. Supplementing with dietary fiber or propionic acid elevates local histamine levels in the gut, rapidly alleviating arthritis in mice. The same effect has been observed in clinical patients [47]. Additionally, supplementing with specific probiotics or prebiotics aims to correct dysbiosis, strengthen the intestinal barrier, and reduce systemic inflammation, thereby indirectly protecting the joints [48]. A balanced diet, regular exercise, and weight management are not only fundamental approaches to managing osteoarthritis but also core strategies for maintaining a healthy gut microenvironment, effectively improving the state of the gut‐brain axis microenvironment at its source. The gut‐joint axis provides a novel systemic intervention strategy for OA and is an important component of the multi‐axis microenvironmental model.

The OA microenvironment is not a single‐factor abnormality but a complex network of multi‐dimensional crosstalk and mutual amplification among immunity‐senescence‐metabolism‐gut‐joint axis. Single‐target interventions can hardly block the pathological cycle, and future therapies must shift to multi‐target, sequential, and personalized synergistic regulation.

## 5 Novel Regenerative Pathway Therapies

As osteoarthritis progresses, articular cartilage and subchondral bone undergo irreversible damage, and traditional treatments can hardly halt the pathological cascade. Regenerative therapies centered on biomaterials and exosomes can repair tissue defects, break pathological vicious cycles, and restore joint microenvironmental homeostasis, achieving a paradigm shift from symptomatic relief to root‐cause repair. This chapter systematically reviews cutting‐edge advances and translational challenges of biomimetic scaffolds, smart hydrogels, exosomes, and their composite systems (Figure 4 and Table 2).

**FIGURE 4:**
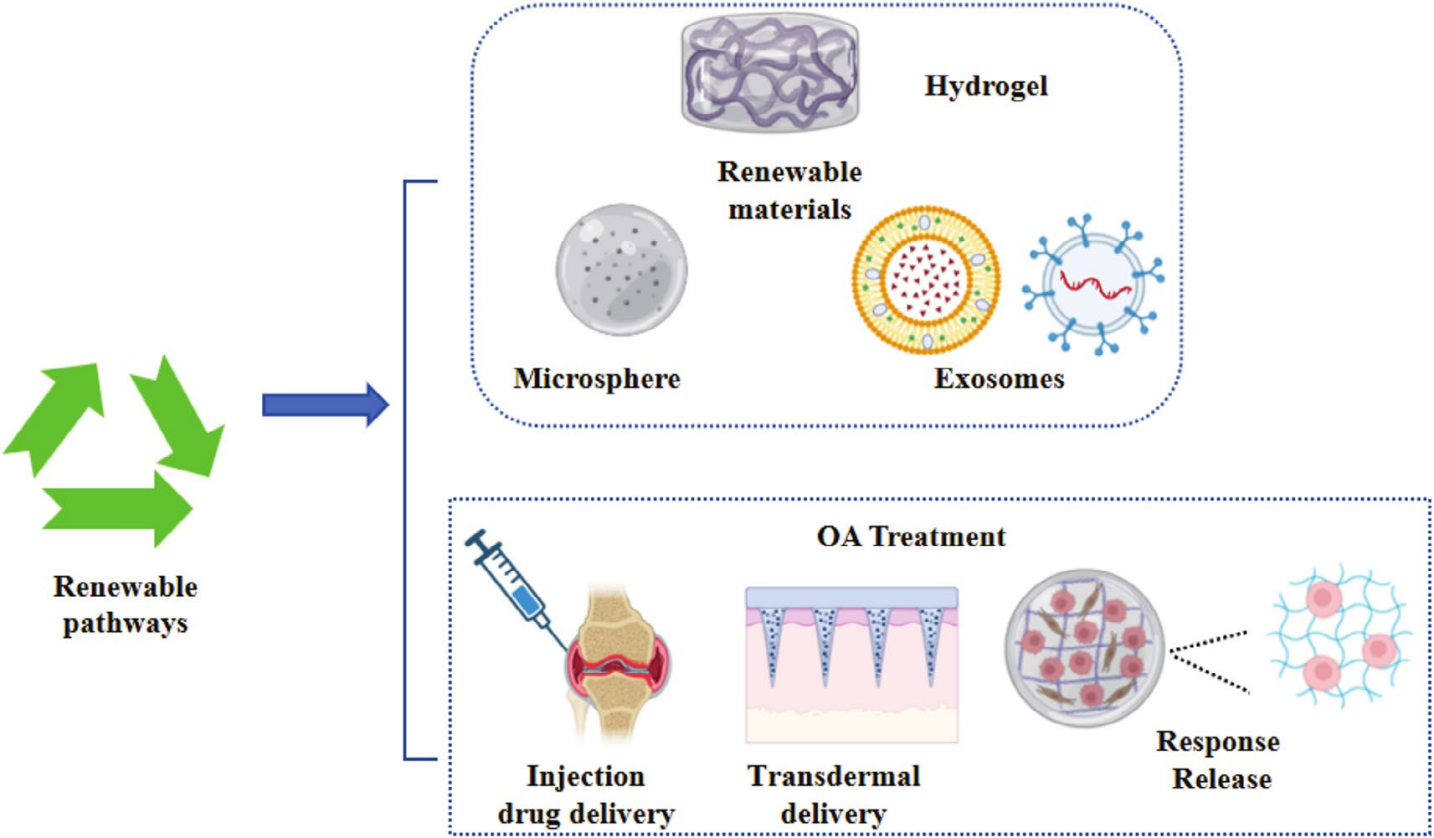
Regenerative therapeutic approaches in OA.

**TABLE 2:** Comparative evaluation of typical biomaterials and exosomes in OA treatment.

Category	Representative materials	Efficacy in OA in vivo models	Targeting ability	Translational stage	Critical limitations	References
Natural biomaterials	Collagen, HA, Chitosan, and so forth.	Good biocompatibility; anti‐inflammatory; cartilage bioaffinity	Low	Clinical	Fast degradation; weak mechanics; unstable in OA microenvironment	[49]
Synthetic polymers	PLGA, PCL, GelMA, and so forth.	Sustained release; good processability; stable structural support	Medium	Clinical	Lack bioactive signals; cannot regulate immune/metabolic axes	[50]
Inorganic materials	HAp, β‐TCP, BG, and so forth.	Effective subchondral bone repair; high mechanical strength	Very low	Clinical	No immune/senescent regulation; poor cartilage integration	[51]
Animal‐derived exosomes	BMSC‐Exo, hUSC‐Exo, and so forth.	Strong chondroprotection; clear mechanism; regulates mitophagy	Medium	Phase I/II	Rapid joint clearance; high cost; difficult standardization	[52]
Plant‐derived exosomes	Spirulina, Epimedium, and so forth.	Moderate efficacy; anti‐inflammatory; high safety	Low	Preclinical	Unclear mechanism; low loading; poor targeting	[53]
Composite systems	Hydrogel+engineered exosomes, and so forth.	Excellent multi‐axis regulation; long retention; sequential release	High	Preclinical	Complex preparation; GMP difficulty; unknown long‐term safety	[54]

### 5.1 Biomaterials

Biomaterials are artificial or natural materials designed to interact with biological systems to assess, treat, enhance, or replace any tissue, organ, or bodily function [55]. Its core characteristics include high biocompatibility without triggering harmful immune responses, controllable degradability with adjustable degradation rates to match tissue regeneration speed, mechanical support, and functional properties such as signal transduction. Based on composition, these materials are categorized into three major types: natural materials, synthetic polymers, and inorganic substances.

#### 5.1.1 Natural Biomaterials

Natural biomaterials such as collagen, hyaluronic acid (HA), chitosan, and decellularized cartilage matrix are widely investigated for OA treatment due to their high biocompatibility, bioactivity, and ability to mimic the native cartilage extracellular matrix. They also offer inherent lubrication, antimicrobial, and pro‐healing properties. However, limitations including weak mechanical strength, rapid degradation, instability in acidic environments, and batch‐to‐batch variability hinder their clinical translation [56]. Current applications in OA range from cartilage defect filling and cell delivery to intra‐articular lubrication, 3D‐printed scaffolds, drug‐loaded microspheres, and bioinks [57]. In mouse DMM/OA models, ROS‐responsive collagen hydrogels scavenge excessive ROS in the pathological microenvironment, inhibiting NLRP3 inflammasome activation and M1 macrophage polarization to reduce matrix degradation. Self‐healing hyaluronic acid hydrogels suppress IL‐1β and TNF‐α via the TLR4/NF‐κB pathway and block THBD‐dependent chondrocyte senescence, achieving dual anti‐inflammatory and anti‐senescence regulation [24]. Addressing the limitations of clinically common HA lubricants—such as poor mechanical properties, rapid degradation, and the need for frequent injections—a self‐healing hydrogel (AHA/CHA) based on dual‐modified hyaluronic acid demonstrates rapid self‐healing, excellent injectability, and cartilage adhesion capacity [58]. It significantly upregulates COL2A1 and ACAN, and SOX9 expression while downregulating MMP3 and MMP13. Simultaneously, it inhibits the THBD pathway to delay chondrocyte senescence [49].

#### 5.1.2 Synthetic Polymers

Synthetic polymers (PLGA, PCL, PEG, GelMA) allow precise control over degradation, mechanics, and drug release kinetics by adjusting monomer ratios and crosslinking density [59]. For example, PLGA degradation can be adjusted from weeks to months by varying its lactide/glycolide ratio, making it suitable for sustained delivery of growth factors or anti‐inflammatory drugs [50]. PCL offers high toughness and slow degradation (2–4 years), rendering it ideal for load‐bearing bone layers in osteochondral scaffolds [60]. PEG derivatives like GelMA form photopolymerizable injectable hydrogels, enabling minimally invasive filling of irregular defects [61]. PCL/nano‐hydroxyapatite scaffolds remodel trabecular architecture and reduce abnormal mechanical loading in rat subchondral sclerosis models, indirectly alleviating cartilage damage [25]. GelMA enables photocurable molding and minimally invasive filling in minipig OA defect models [62]. However, synthetic materials lack bioactive recognition signals and cannot actively regulate the immune‐senescence‐metabolic multi‐axis microenvironment, requiring functionalization to match OA pathology.

#### 5.1.3 Inorganic Biomaterials

Inorganic biomaterials (HAp, β‐TCP, bioactive glass) are key carriers for subchondral bone repair, but their performance in OA‐specific in vivo models differs drastically. In rat DMM OA models, HAp improves subchondral bone mass and mechanical properties but degrades too slowly and lacks immunomodulatory capacity, failing to inhibit synovitis or chondrocyte senescence. It only alleviates sclerosis without halting cartilage degeneration [51]. In rabbit subchondral cyst OA models, β‐TCP degrades in line with bone regeneration but is brittle and non‐chondrophilic, unable to penetrate deep cartilage zones, making integrated osteochondral repair unachievable [63]. In mouse inflammatory OA models, BG releases Si^4+^ to promote osteogenesis via MAPK/ERK but dissolves rapidly in high ROS/MMP OA microenvironment and lacks targeting, leading to fast clearance [64, 65]. Overall, Inorganic materials only repair bone structural defects and cannot regulate the immune‐senescence‐metabolic‐inflammatory multi‐axis imbalance in OA; they cannot achieve disease modification as monotherapy. Combination with hydrogels, exosomes, or anti‐inflammatory drugs is mandatory to fulfill both bone repair and microenvironment regulation.

### 5.2 Extracellular Vesicles

Extracellular vesicles (EVs) are spherical or oval‐shaped vesicles released into the extracellular space following the rupture of cell membranes. They are secreted by cells and carry bioactive molecules from within the cell [66]. Exosomes are classified into animal‐derived exosomes and plant‐derived exosomes. In recent years, exosomes have demonstrated promising application trends in osteoarthritis treatment research. By delivering functional molecules, they promote cartilage repair, suppress synovial inflammatory responses, modulate immune cell activity, and participate in subchondral bone remodeling, thereby delaying the progression of joint degeneration. Currently, they are being actively explored as potential therapeutic tools or drug delivery vehicles.

#### 5.2.1 Animal‐Derived Exosomes

Animal‐derived exosomes are nanoscale extracellular vesicles (30–150 nm in diameter) that play a key role in intercellular communication. Composed of a lipid bilayer, they carry bioactive cargo such as proteins, lipids, and nucleic acids, offering low immunogenicity, high biosafety, and strong tissue permeability enabling deep cartilage penetration [67]. In OA, lipid metabolism dysregulation contributes to cartilage degeneration. High miR‐140 loading targets CAPN1 to restore mitophagy; strong cartilage protection in mouse/rat OA models, but joint half‐life < 6 h [52]. Additionally, engineered exosomes enable precise therapeutic delivery. miR‐140‐loaded exosomes from human urinary stem cells (hUSCs‐140‐Exos) improve gait and subchondral bone architecture in OA models by activating the miR‐140/CAPN1‐mitophagy axis [26]. Similarly, exosomes from human amniotic epithelial stem cells (hAESCs‐Exo) deliver lncRNA ACTA2‐AS1 to inhibit chondrocyte ferroptosis by suppressing ACSL4, thereby protecting cartilage [68]. However, they exist critical bottlenecks. Rapid clearance after intra‐articular injection leads to short retention time, large‐scale production is costly and difficult to standardize, and targeting mainly depends on passive accumulation without precise homing. Only a few have entered Phase I/II clinical trials, and translation is limited by scalable manufacturing and quality control.

#### 5.2.2 Plant‐Derived Exosomes

Plant‐derived exosomes (PENs) are nanoscale vesicles secreted by plant cells, carrying lipids, proteins, nucleic acids, and bioactive molecules. They facilitate interspecies communication and can modulate mammalian cellular functions. Compared to animal exosomes, PENs typically have a larger size, contain unique plant‐derived components, wide availability, ultra‐low cost, and negligible immunogenicity [53]. In OA, where dysregulated energy metabolism drives progression, PEN‐based systems show therapeutic potential. A composite hydrogel (Rh Gel@SP‐EVs) combining rhein‐based gel with extracellular vesicles from Spirulina platensis was shown to mitigate mitochondrial dysfunction, correct cartilage matrix metabolism, and inhibit the JAK/STAT3 pathway, effectively alleviating OA in mouse models with excellent biocompatibility [27]. For bone defects in OA, a 3D GelMA hydrogel loaded with exosomes from Epimedium GelMA@Exo enhanced exosome retention and sustained release. This system promoted osteoblast proliferation, migration, and mineralization, reduced senescence, and stimulated angiogenesis via upregulation of osteogenic markers and activation of the PI3K/Akt pathway, but targeting relies on passive accumulation [69]. Similarly, engineered exosome‐like nanovesicles from water celery (WELNs), functionalized with chondrocyte‐targeting peptides, restored mitochondrial function, maintained chondrocyte phenotype, suppressed inflammation, and reversed cartilage degeneration in OA models [70]. However, they also have clear disadvantages. Cargo loading efficiency is much lower than that of animal‐derived exosomes, targeted modification is challenging, and mechanisms remain incompletely understood. Most are still at the preclinical stage without systematic safety and efficacy validation, far from clinical application.

### 5.3 Comprehensive Applications

Taken together, monotherapy with either biomaterials or exosomes has inherent limitations in osteoarthritis treatment: although biomaterials provide structural support and drug delivery platforms, they lack bioactive signals for actively regulating the pathological microenvironment; exosomes exhibit excellent cartilage repair and immunomodulatory functions but are rapidly cleared in vivo with insufficient targeting, failing to exert long‐term effects. Therefore, the construction of an integrated composite delivery system by combining biomaterials and exosomes enables complementary advantages and synergistic efficacy, representing the most promising strategy to overcome current therapeutic bottlenecks and achieve precise regulation of the multi‐axis microenvironment [58, 71].

Composite delivery systems combining biomaterials and exosomes exhibit complementary advantages and represent the most translation‐promising strategy for OA regenerative therapy. Biomaterial carriers not only markedly prolong the retention of exosomes in the joint cavity but also enable spatiotemporally controlled release matching OA pathological progression through multi‐responsive design to ROS, pH, and matrix metalloproteinases [54]. Modification with cartilage‐targeting peptides further improves lesion‐site enrichment and enhances local therapeutic efficacy [72]. Sequential hierarchical delivery systems (first releasing anti‐inflammatory exosomes to control inflammation, then delivering pro‐regenerative exosomes to promote cartilage repair) achieve full‐cycle dynamic regulation of the immune‐senescence‐metabolic multi‐axis microenvironment [73]. In animal models, these systems simultaneously suppress inflammation, eliminate senescent cells, restore cartilage matrix anabolism, and improve subchondral bone architecture [74, 75].

Nevertheless, current composite systems still face challenges such as unstable cargo loading uniformity, unvalidated long‐term in vivo safety, and difficulty in meeting GMP‐standard manufacturing. Further optimization of material‐exosome interfacial compatibility, controlled‐release logic, and targeting strategies is required to better adapt to the complex and dynamic OA microenvironment and accelerate clinical translation.

## 6 Challenges and Outlook

OA is a complex degenerative disease driven by multi‐axis microenvironmental imbalance, involving an interactive network of immune dysregulation, cellular senescence, metabolic disorders, and cross‐organ regulation via the gut–joint axis. Current mainstream treatments only provide symptomatic relief or structural replacement without dynamically correcting the pathological microenvironment. Although regenerative therapies offer disruptive potential, they still face core challenges including high microenvironmental heterogeneity, material compatibility, exosome standardization, multi‐target synergy, and insufficient clinical translation. Future therapies must be guided by the multi‐axis microenvironmental imbalance model toward intelligent, personalized, and sequential precision treatment. Current mainstay treatments: NSAIDs and surgery only offer symptomatic relief or are highly invasive, failing to address the disease's dynamic and heterogeneous nature. The evolving microenvironment requires temporally sequenced interventions, which existing monotherapies cannot provide. Moreover, achieving multi‐target synergy remains challenging due to the coexistence of diverse pathological factors. Regenerative strategies also face significant hurdles. Natural biomaterials often suffer from poor mechanical strength and rapid degradation; synthetic polymers, while tunable, lack bioactive signaling; and inorganic materials are too brittle for cartilage. Exosome therapies, though promising, are limited by short joint retention, rapid clearance, variable composition due to non‐standardized production, and high costs for GMP‐scale manufacturing.

To address these challenges, future interdisciplinary integration will drive innovation in smart materials, with multifunctional integration and intelligent responsiveness becoming key development directions. Leveraging nanotechnology, 3D printing, and synthetic biology, smart materials capable of specifically responding to OA microenvironment signals can be engineered. These materials enable on‐demand, time‐controlled drug delivery. Examples include the MMP13 enzyme‐responsive peptide chains for precise drug release mentioned in the document, as well as spatiotemporal controlled‐release systems that first release anti‐inflammatory exosomes, followed by pro‐repair exosomes, representing this cutting‐edge direction. Simultaneously, deepening microenvironment mechanism research and personalized diagnostics/therapeutics—leveraging multi‐omics technologies to analyze microenvironment characteristics across different OA subtypes and disease stages—will aid in identifying novel key therapeutic targets. This will enable the development of biomarker‐based diagnostic tools for classification, ultimately achieving personalized precision medicine and tailoring combination therapies for individual patients. Furthermore, engineered exosomes—modified through genetic engineering to overexpress specific therapeutic miRNAs or surface‐modified with targeting peptides—can significantly enhance targeting precision and therapeutic efficacy. Furthermore, exploring novel therapeutic dimensions—such as deepening research into systemic mechanisms like the gut‐joint axis—opens new avenues for OA treatment. Modulating gut microbiota, intervening in bile acid metabolism, or utilizing GLP‐1 receptor agonists can regulate joint inflammation and metabolism at the systemic level. Furthermore, strengthening clinical translation and standardization efforts will advance the establishment of international standards and quality control systems for exosomes and biomaterials. Concurrently, more rigorously designed clinical trials are needed to validate the safety, efficacy, and long‐term therapeutic outcomes of these novel regenerative strategies in human subjects.

In summary, the treatment paradigm for osteoarthritis is undergoing a fundamental shift. Future success will depend on a deep understanding of the joint microenvironment's complexity. Leveraging interdisciplinary technologies, we must develop next‐generation regenerative material therapies capable of dynamic response, multi‐targeted synergy, and integrating regenerative capacity with intelligent regulation. This will ultimately enable the transition from disease delay to functional reversal.

## 7 Conclusion

OA is not a simple mechanical wear disease, but a systemic homeostatic collapse centered on multi‐axis microenvironmental imbalance of immunity–senescence–metabolism–gut–joint axis. Traditional treatments only relieve symptoms without blocking pathological cycles; regenerative strategies centered on renewable biomaterials and engineered exosomes achieve synergistic effects of anti‐inflammation, anti‐aging, metabolic correction, and tissue repair by targeting the multi‐dimensional interactive network, driving the upgrade of OA treatment from symptomatic management to root‐cause disease modification. Composite systems of biomaterials and exosomes overcome the bottlenecks of monotherapy, enabling intelligent responsiveness, targeted delivery, and sequential regulation, representing the core direction of next‐generation OA therapy. Despite remaining challenges in material optimization, exosome standardization, and clinical translation, the deep integration of interdisciplinary disciplines will bring precise, intelligent, and personalized regenerative therapies to new hope for OA patients worldwide.

## Author Contributions

**Shenghua Wu:** writing – original draft, supervision. **Quanchun Liu:** writing – original draft, supervision. **Yongbiao Meng:** writing – review and editing, project administration.

## Funding

This work was supported by The Science and Technology Program of Traditional Chinese Medicine in Zhejiang Province (2024ZF127).

## Ethics Statement

The authors have nothing to report.

## Consent

The authors have nothing to report.

## Conflicts of Interest

The authors declare no conflicts of interest.

## Data Availability

The data that support the findings of this study are available on request from the corresponding author. The data are not publicly available due to privacy or ethical restrictions.
